# Caregivers' Loss of the Dyadic Experience After Their Care Partner's Death

**DOI:** 10.1093/geroni/igab046.1222

**Published:** 2021-12-17

**Authors:** Harleah Buck

**Affiliations:** University of Iowa, Iowa City, Iowa, United States

## Abstract

One emerging dyadic concept is the experience of family caregivers when their care partner dies and their dyadic relationship comes to an end. This study qualitatively examined and characterized the loss of the dyadic experience for the caregiver after the death of their care partner. Data was accrued as part of a randomized clinical trial in 29 older hospice caregivers. Iterative thematic analysis focused on dyadic processes before, during and post death. Using two relational parameters from Relational Turbulence Theory resulted in a preliminary characterization of a new concept - dyadic dissolution as a cognitive and affective process whereby a remaining member of a dyad experiences relational uncertainty and partner interference while adapting (or not) to the death of their care partner. Findings suggest that asking several open-ended questions about the dyadic relationship will enable assessment for any continuing impact of relational uncertainty and partner interference on bereaved caregivers.

